# The combination of early treatment response and ypT stage is a novel metric to stage rectal cancer patients treated with neoadjuvant chemoradiotherapy

**DOI:** 10.18632/oncotarget.14708

**Published:** 2017-01-17

**Authors:** Jian Cui, Lin Yang, Lei Guo, Yongfu Shao, Dongfeng Tan, Ni Li, Haizeng Zhang

**Affiliations:** ^1^ Department of Pathology and Laboratory Medicine, The University of Texas M. D. Anderson Cancer Center, Houston, TX, USA; ^2^ Department of Pathology, National Cancer Center/Cancer Hospital, Chinese Academy of Medical Sciences and Peking Union Medical College, Beijing, China; ^3^ National Office for Cancer Prevention and Control, National Cancer Center/Cancer Hospital, Chinese Academy of Medical Sciences and Peking Union Medical College, Beijing, China

**Keywords:** rectal cancer, neoadjuvant chemoradiotherapy, tumor regression grade, ypT stage, TNM staging system

## Abstract

Rectal cancer patients receiving neoadjuvant chemoradiotherapy (NCRT) are currently classified using the same Tumor-Node-Metastasis staging system as those patients without NCRT. We determined whether the combination of tumor treatment response (TRG) and ypT stage more accurately assesses primary tumors in rectal cancer after NCRT. We analyzed data from 329 rectal cancer patients treated with NCRT followed by radical resection. Cox proportional hazards models were used to evaluate the effects of different staging parameters on disease-free survival (DFS). ypN stage and TRG were independently associated with 3-year DFS, but ypT stage was not. We developed a new modified T stage classification metric (M-TTRG) that categorized patients into 5 subgroups based on ypT stage and TRG, with weighting by β-coefficients from multivariate analyses. The incidence of patients developing local or distant recurrence increased with increasing M-TTRG level. All five M-TTRG classes correlated with 3-year DFS. Improvement was seen in the model with M-TTRG classification compared with ypT stage, based on area under the curve after computing receiver operating characteristic curves. Our modified ypTNM staging system significantly improved prediction of 3-year DFS. This suggests TRG could complement ypT stage, and we propose the new M-TTRG metric could be used to better classify NCRT-treated patients, thereby improving treatment and assessing prognosis. The M-TTRG metric might be applicable to other types of cancer.

## INTRODUCTION

Neoadjuvant chemoradiotherapy (NCRT), also known as preoperative CRT, aims to downstage and downsize tumors to enhance the curative resection rate. NCRT is now the standard treatment for patients with locally advanced mid-low rectal cancer [1, 2]. The American Joint Committee on Cancer (AJCC) Tumor-Node-Metastasis (TNM) staging system evaluates patient prognosis with a prefix ”y” and employs the same category definitions for rectal cancer patients with and without NCRT. Previous studies have suggested that the current AJCC staging system cannot precisely assess prognosis or survival for patients after NCRT, especially in certain subgroups [3]. Therefore, the current AJCC staging system should be modified as accurate restaging after NCRT could help to improve postoperative prognosis evaluation and adjuvant treatment prescription for patients with rectal cancer after NCRT.

In most rectal cancer patients after NCRT, the tumors regress to some extent, and the ypT stage might change accordingly, resulting in inadequate evaluation of the tumor invasion status. The definition of the postoperative ypT stage only focuses on the invasion depth of the primary tumor but the degree of the treatment response following NCRT (e.g., assessment of scars, fibrotic areas or cellular mucinous lakes, etc.) is not considered. Tumor regression grade (TRG) is a semi-quantitative scoring system that evaluates the degree of remnant tumor, informing on the tumor response to NCRT. Previous studies have suggested that TRG is a useful prognostic factor that correlates with disease free survival (DFS). The 5-year DFS after NCRT and curative resection was 86% for complete response, 75% for intermediate pathologic response, and 63% for no/minor regression, suggesting that TRG assessment should be implemented in pathologic evaluation and prospectively validated in further studies [4]. Since the ypT stage focuses on the invasion depth of the primary tumor while TRG reflects the degree of treatment response, we hypothesized that the combination of ypT stage and TRG of the primary tumor might yield an improved assessment of prognosis. Here, we developed a modified T stage classification metric (M-TTRG) and assessed its prognostic value by analyzing data from 329 patients with rectal cancer after NCRT.

## RESULTS

### Patient and tumor characteristics

All the 329 rectal cancer patients in this study were recruited from the Cancer Institute and Hospital, Chinese Academy of Medical Sciences (CICAMS) between September 1994 and December 2013. Characteristics and clinical information for all the patients were shown in Table 1. Among the patients, 226 (68.7%) were males and 103 (31.3%) were females, with a median age of 55 years (range = 27-81 years). Before NCRT, 221 (67.2%) patients were in T3 stage while 108 (32.8%) were in T4 stage, with 265 (80.5%) of tumors being cN+ according to preoperative evaluation. The numbers of patients who underwent LAR, APR and Hartmann resection procedures were 138 (41.9%), 170 (51.7%) and 21 (6.4%), respectively. Histological assessment of primary tumors revealed no viable tumor (TRG1, pCR) in 46 (14.0%) patients. On the other hand, 55 (16.7%) patients were classified as TRG2, 127 (38.6%) as TRG3, 68 (20.7%) as TRG4, and 33 (10%) as TRG5. After surgery, 157 (47.7%) patients were treated with adjuvant chemotherapy. The median follow-up time was 37.3 months (range = 12.2-202.5 months), with eight (2.4%) patients developing local recurrence, 80 (24.3%) developing distant metastasis, and five (1.5%) developing both.

**Table 1:** Characteristics of 329 Patients with Rectal Cancer Treated with NCRT

	Total (%)	*P*
Age		0.6952
≤ 60	232 (70.5)	
> 60	97 (29.5)	
Gender		0.9285
Male	226 (68.7)	
Female	103 (31.3)	
Distance from the anal verge		0.1139
≥ 5 cm	91 (27.7)	
< 5 cm	238 (72.3)	
Surgical Procedure		0.6505
LAR	138 (41.9)	
APR	170 (51.7)	
Hartmann resection	21 (6.4)	
Clinical T stage		0.6185
cT3	221 (67.2)	
cT4	108 (32.8)	
Clinical N stage		0.4734
cN0	64 (19.5)	
cN+	265 (80.5)	
Interval completion of NCRT to surgery		0.1187
≥ 7 weeks	177 (46.2)	
< 7 weeks	152 (53.8)	
Concurrent chemotherapy		
Capecitabine	136 (41.3)	0.5056
Capecitabine plus oxaliplatin	193 (58.7)	
Adjuvant chemotherapy		0.4651
Yes	157 (47.7)	
No	172 (52.3)	
ypTNM		<0.0001
0	41 (12.5)	
I	54 (16.4)	
II	99 (30.1)	
III	135 (41.0)	
Follow-up		-
Local recurrence	8 (2.4)	
Systematic recurrence	80 (24.3)	
Local and systematic recurrence	5 (1.5)	

Abbreviations: LAR, low anterior resection; APR, abdominoperineal resection; NCRT, neoadjuvant chemoradiotherapy. P value was calculated by Kaplan-Meier survival analysis correlated with 3-year DFS.

### Impacts of ypTNM stage and TRG on patient DFS

After evaluation of the postoperative ypTNM stage, the downstaging effect of NCRT on primary tumors was observed in 172 (52.3%) patients and ypTNM correlated with 3-year DFS (*P* < 0.0001; Table 2). TRG was found to be a prognostic factor for DFS by univariate analysis (*P* < 0.0001). Forty-five of 46 (97.8%) patients achieving pCR (TRG1) experienced 3-year DFS. All of these 46 TRG1 patients were excluded from further statistical analyses.

**Table 2:** Univariate Analysis of Pathological Factors of 3-year DFS

	No.*	3-year DFS rate	*P*
ypT stage			0.2272
ypT1	8	75.0%	
ypT2	65	80.0%	
ypT3	181	65.6%	
ypT4	29	60.6%	
TRG			<0.0001
TRG2	55	82.7%	
TRG3	127	73.0%	
TRG4	91	61.0%	
TRG5	10	15.0%	
ypN			<0.0001
ypN0	152	79.9%	
ypN1	92	56.2%	
ypN2	39	53.2%	
Modified ypT-TRG (M-TTRG) classification			<0.0001
M-TTRG1	91	83.9%	
M-TTRG2	81	68.8%	
M-TTRG3	91	59.7%	
M-TTRG4	20	33.3%	

Abbreviations: DFS, disease free survival; TRG, tumor regression grade.*46 patients achieving pCR in primary tumor were excluded and 283 patients were included in the analysis.

**Figure 1:**
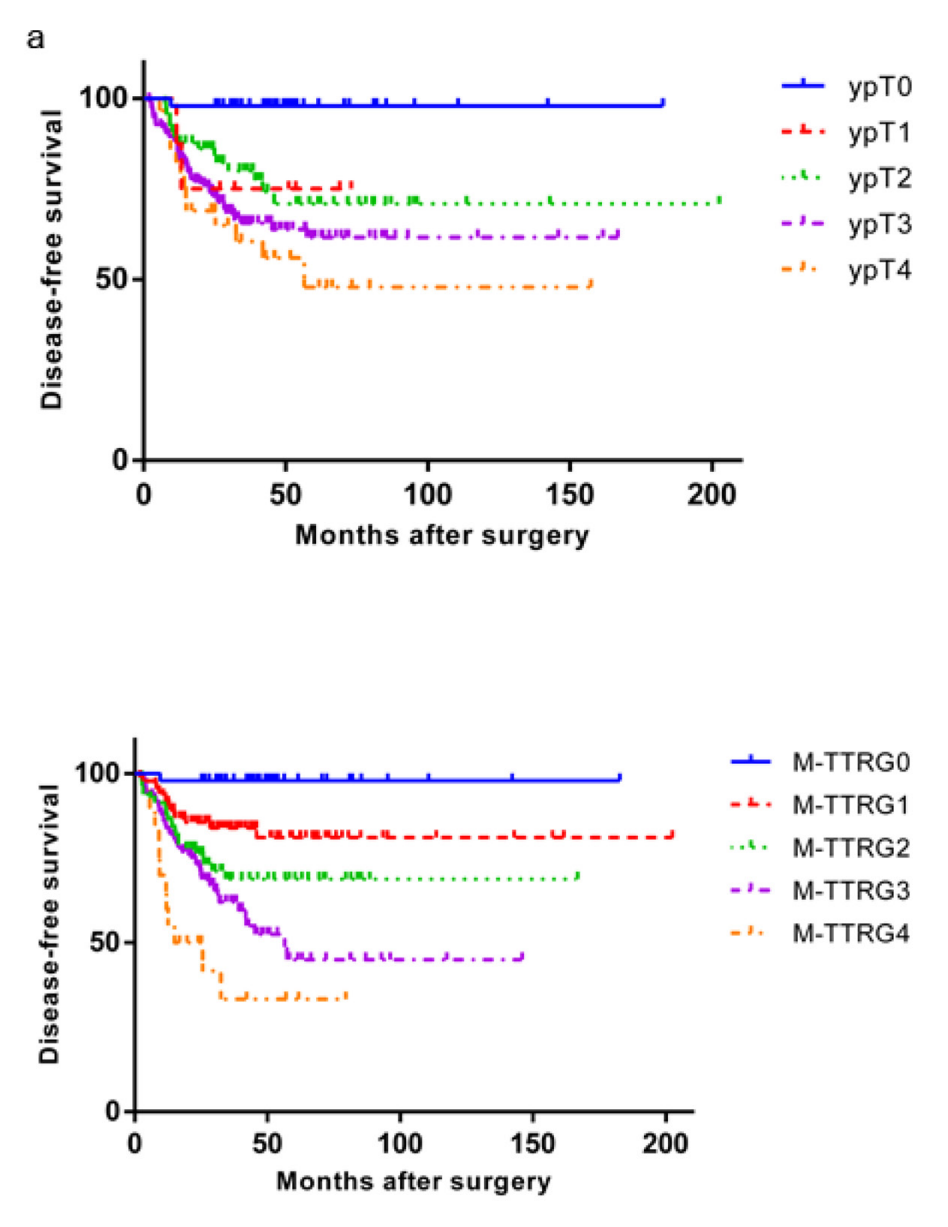
Kaplan-Meier Analysis of Disease Free Survival of Rectal Cancer Patients Treated with NCRT and Surgery According to the ypT Stage and M-TTRG Classification

Considering only ypT stage, eight (2.8%), 65 (23.0%), 181 (64.0%) and 29 (10.2%) patients were in ypT1, ypT2, ypT3 and ypT4 stage, respectively. However, ypT stage was not correlated with 3-year DFS by univariate analysis (*P* = 0.2272; Figure 1a). On the other hand, 152 (53.7%), 92 (32.5%), and 39 (13.8%) patients were in ypN0, ypN1 and ypN2 stage, respectively, and ypN correlated with 3-year DFS (*P* < 0.001). We then classified the patients by lymph node metastasis status. Within the ypN0 subgroup without lymph node metastasis, ypT stage did not correlate with DFS (*P* = 0.266). However, TRG was a significant prognostic factor for 3-year DFS in patients without lymph node metastasis (*P* = 0.037). In patients with lymph node involvement (ypN1 and ypN2), ypT stage did not correlate with 3-year DFS (*P* = 0.682) while TRG did (*P* < 0.001). We then conducted multivariate analyses of these factors and identified that ypN stage (*P* < 0.0001) and TRG (*P* < 0.0001), but not ypT stage (*P* = 0.2171), were two independent factors for 3-year DFS.

### Modified ypT stage in combination with TRG

**Figure 2:**
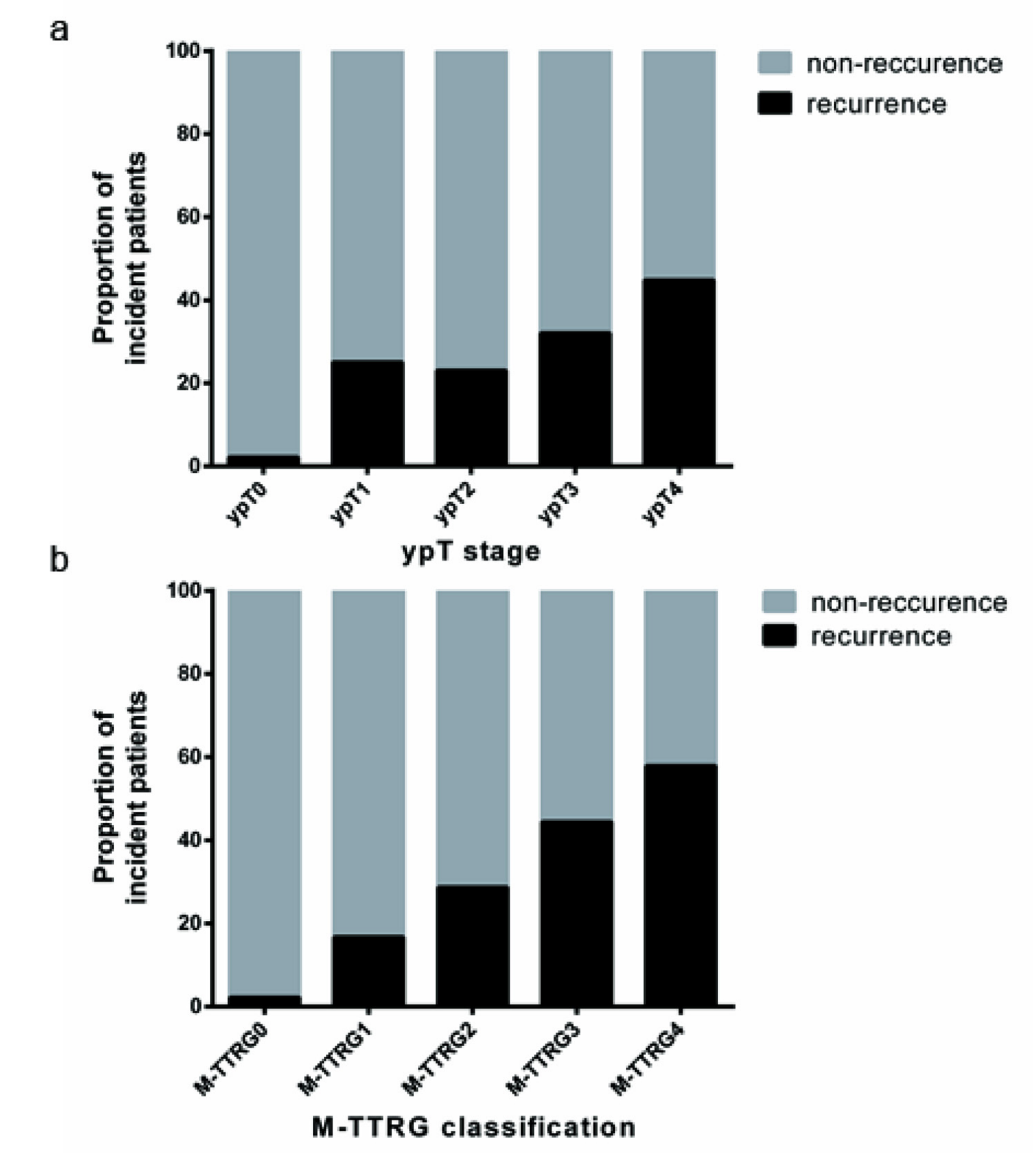
Distribution of Patients Developing Local/System Recurrence According to the ypT Stage and M-TTRG Classifications

**Figure 3:**
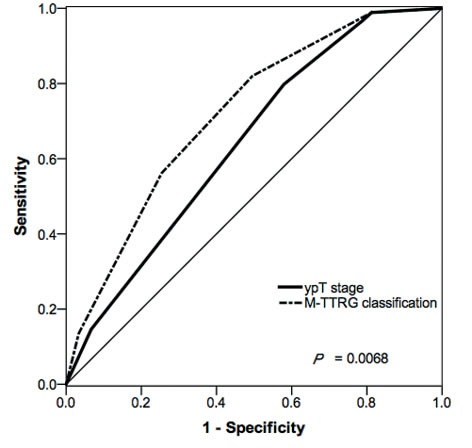
Comparison of ROC between ypT Stage and M-TTRG Classification in Evaluating Local/Distant Metastasis The straight line indicates random classification. The areas under the ROC (AUC) are based on logistic regression models incorporating the ypT stage and M-TTRG classifications.

ypT stage and TRG were two factors to describe primary tumor depth and tumor remnants, respectively. Therefore, Table 3 proposes a modified primary tumor staging M-TTRG classification, which is the sum of ypT stage and TRG weighted by β-coefficients, calculated by multivariate analysis. Given the distribution of scores, we divided the M-TTRG classified patients into five subtypes with 46 (14.0%), 91 (27.7%), 81 (24.6%), 91 (27.7%) and 10 (6.1%) patients assigned to M-TTRG0, M-TTRG1, M-TTRG2, M-TTRG3 and M-TTRG4 groups, respectively. The number of patients developing local or distant recurrence increased with increasing M-TTRG classification but not with increasing ypT stage (Figure 2). Our five-class M-TTRG correlated with 3-year DFS (P < 0.0001, Figure 2b). To assess the discriminative improvement after adding TRG to the ypT stage, we measured the area under the curve (AUC) for ypT stage and M-TTRG classifications, which were 0.641 and 0.719, respectively. Therefore, improvement in AUC was seen between the models with or without TRG (*P* = 0.0068; Figure 3).

**Table 3:** Weight Assignments for the ypT stage and TRG Scoring Systems

Modified ypT stagea	ypT stage	TRG	Weighted score
M-TTRG0	T0	TRG1	0.604
M-TTRG1	T1	TRG2	1.576
	T2	TRG2	1.944
	T1	TRG3	2.180
	T3	TRG2	2.312
	T2	TRG3	2.548
	T4	TRG2	2.680
	T1	TRG4	2.784
M-TTRG2	T3	TRG3	2.916
M-TTRG3	T2	TRG4	3.152
	T4	TRG3	3.284
	T1	TRG5	3.388
	T3	TRG4	3.520
M-TTRG4	T2	TRG5	3.756
	T4	TRG4	3.888
	T3	TRG5	4.124
	T4	TRG5	4.492

a M-TTRG classification: modified ypT stage by combining ypT stage and TRG; b Estimated β-coefficients for every increase in ypT and TRG were 0.368 and 0.604. Total weight was calculated by the sum of increased β of each ypT and TRG.

### Modified ypTNM stage using M-TTRG classification

After multivariate analysis including M-TTRG classification and ypN stage, we identified that these two factors were independently associated with 3-year DFS (*P* < 0.001). After stratification by lymph node metastasis status, all M-TTRG classifications were associated with 3-year DFS (*P* < 0.001) within the subset of ypN0 and ypN1-2 patients. We further combined M-TTRG stage with ypN stage and identified significant differences in 3-year DFS among patients classified using the modified ypTNM staging system (*P* < 0.001, Table 4).

**Table 4:** Modified ypTNM staging system with M-TTRG classification

Modified ypTNM Stage	M-TTRG	ypN	No. of patients	3-year DFS rate	*P*
0	M-TTRG0	N0	42	97.6%	<0.0001
I	M-TTRG1 M-TTRG2	N0	99	84.6%	
IIA	M-TTRG3 M-TTRG4	N0	53	71.3%	
IIB	M-TTRG0 M-TTRG1 M-TTRG2	N1	56	65.3%	
IIIA	M-TTRG0 M-TTRG1 M-TTRG2	N2	38	47.7%	
IIIB	M-TTRG3 M-TTRG4	N1	21	42.6%	
IIIC	M-TTRG3 M-TTRG4	N2	20	38.9%	

Abbreviations: DFS, disease free survival; M-TTRG classification: modified ypT stage by combining ypT stage and TRG.

## DISCUSSION

In this study, we modified the ypTNM stage by replacing the ypT stage with our newly developed M-TTRG classification and found that 3-year DFS significantly differed among patients in different modified stages. The ypT stage only indicated the depth of primary tumor invasion and TRG was an indicator of the percentage of viable primary tumor. Therefore, we treated TRG as a complement to ypT stage when evaluating the primary tumor after NCRT. This highlighted the usefulness of the M-TTRG classification, which combines the ypT stage and TRG in the evaluation of primary rectal cancer treated by NCRT.

The TNM staging system is important in clinical decision-making, and the outcome of the patients within each TNM stage varies significantly [5]. Postoperative staging is performed using the same system for patients treated with and without NCRT. However, after NCRT, tumor regression might result in inadequate assessment in primary tumor. As expected, in this study, the likelihood of 3-year DFS decreased with increased ypTNM stage. However, the ypTNM stage could roughly classify NCRT-treated patients mainly because the key determination was lymph node involvement, whereas the depth of tumor invasion had less impact on DFS [6–9]. In addition, patients with pCR had the most favorable outcome, which might overestimate the difference among a large number of patients with residual disease. We identified that TRG and lymph node metastasis status were two independent predictors of 3-year DFS. Regardless of lymph node status, TRG could predict prognosis well whereas ypT stage could not. We demonstrated an increasing risk of recurrence or metastasis with higher TRG stage, which was consistent with previous results reporting TRG as an independent prognostic factor for patients suffering from various types of cancer and treated with NCRT [10–15]. After we replaced ypT stage with M-TTRG classification in the current TNM staging system, both the M-TTRG and ypN stages also displayed good prognostic power for 3-year DFS.

Several other studies evaluating the impact of NCRT also showed that the current staging system could not precisely assess prognosis. It was reported that four-grade risk classification, i.e., classifying patients into low (ypT0-isN0, ypT1N0, ypT2N0), intermediate (ypT0-2N1, ypT3N0), moderate (ypT0-2N2, ypT3N1, ypT4N0), and high (ypT3N2, ypT4N1-2) risk groups, could more precisely reflect survival outcomes of patients after NCRT than ypStage [3, 16]. Rizk *et al* found that lymph node status and distant metastasis were two useful prognostic factors for patients with esophageal adenocarcinoma who received NCRT before esophagectomy, whereas the depth of tumor invasion and estimated treatment response had less impact on survival [17]. Swisher *et al*. incorporated the extent of the pathologic response into the ypTNM staging system and proposed a revised staging system that better predicted the outcome of esophageal cancer patients following NCRT [18].

Here, for the first time, we systemically evaluated the prognostic value of primary tumor characteristics by combining the tumor invasion depth (ypT stage) and early treatment response (TRG) for rectal cancer patients after NCRT. Our findings agreed with standardized re-evaluation of surgical specimens by two blinded gastrointestinal pathologists, suggesting that our results are reliable. Furthermore, with a median follow-up of over 3 years, we recalculated the 3-year DFS according to our modified TNM stage to validate the feasibility of the methodology, and each subgroup exhibited a more accurate survival rate. The modified ypT stage, namely M-TTRG classification, was not a simple combination of the ypT stage and TRG, but was weighted by the *β*-coefficients obtained from multivariate analyses. Thus, our study could provide a model for the precise prognosis of rectal cancer patients after NCRT followed by curative resection and might be applicable to other types of cancer.

This study has certain limitations due to its retrospective nature. First, patients were recruited in a single center over a long time period. But the majority patients (89%) underwent radical surgery following NCRT between 2004 and 2013. Given that only a small number of patients were available for some TNM and TRG groups, we merged two adjacent subgroups to obtain better results. Second, the chemotherapy regimens administered to the patients were heterogeneous and not all the patients received adjuvant chemotherapy. Third, tumor treatment response after NCRT includes not only the primary tumor regression grade but also lymph nodes regression grade [19]. Most studies, including ours, focused on tumor treatment response in primary tumor. Therefore, our proposed TNM staging system was based on preliminary results. In the future, prospective, multicenter, randomized clinical trials on larger cohorts should be conducted to further substantiate our findings. Nonetheless, we have shown that the combination of depth of tumor invasion (ypT stage) and the percentage of viable tumor remaining (TRG) can more accurately predict the prognosis of patients treated with NCRT in terms of primary tumor compared with the current staging system. Our proposed modified ypTNM staging system enables a more precise subclassification of NCRT-treated patients with rectal cancer. We suggest that the early treatment response should be incorporated into the ypTNM staging system to better predict the outcome of these patients.

## MATERIALS AND METHODS

### Patients

We performed a retrospective study of 329 patients with rectal cancer recruited from the Cancer Institute and Hospital, Chinese Academy of Medical Sciences (CICAMS) between September 1994 and December 2013. This study was approved by the institutional review board of CICAMS. Preoperative stages were determined by endorectal ultrasound, abdominal-pelvic CT and/or pelvic MRI according to the 7th edition of the AJCC Cancer Staging system [20].

The inclusion criteria for patients in this study were: (1) biopsy-proven adenocarcinoma; (2) the inferior edge of the tumor was located less than 10 cm from the anal verge; (3) clinical stage II to III, and (4) the patient received preoperative NCRT followed by radical surgery. Also, none of the 329 patients had any of the following: (1) inflammatory bowel disease, including ulcerative colitis and Crohn's disease; (2) hereditary colorectal cancer, including familial adenomatous polyposis (FAP) and hereditary non-polyposis colorectal cancer (HNPCC); (3) clinical evidence of distant metastatic disease, including lateral lymph node metastases; or (4), a second malignant tumor found pre-operation, post-operation or intra-operation. Characteristics and clinical information for all the patients were obtained from medical records.

Preoperative chemoradiotherapy and surgical resection

Patients underwent a long course of preoperative radiotherapy with total doses of ~40-50.67Gy (median 50 Gy) in 20 to 28 fractions delivered directly to the tumor and the regional pelvic lymph nodes, concurrently with capecitabine with or without oxaliplatin (Table 1). Radical surgeries including low anterior resection (LAR), abdominoperineal resection (APR) or the Hartmann procedure according to the total mesorectal excisions (TME) principle, were performed within a median interval of 6 weeks after NCRT. All patients were recommended to receive adjuvant chemotherapy regardless of the surgical pathological results.

### Pathologic analysis

**Figure 4:**
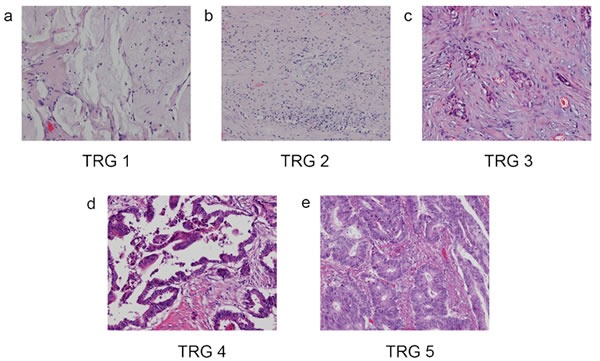
Examples of TRG **A**. TRG 1: Complete regression with no residual tumor cell. **B**. TRG 2: Rare residual tumor cell. **C**. TRG3: Fibrosis outgrown by residual tumor cell. **D**. TRG 4: Residual tumor cell outgrown by fibrosis. **E**. TRG 5: Absence of regression with no fibrosis.

All the patients were staged according to the 7th edition of the AJCC Cancer Staging System for rectal cancer. The hematoxylin-eosin stained slides of the primary tumors and regional lymph nodes were reviewed and independently confirmed by two gastrointestinal pathologists, who were blinded to the study design. TRG was used to evaluate the response of patients with rectal cancer to NCRT. The extent of residual primary tumor was assessed by estimating the percentage of residual tumor cells in the total abnormal area, which included tumor cells, ulcer, fibrotic areas, acellular mucinous lakes, degenerative/necrotic areas and areas of inflammation. The percentage of viable residual tumor was designated as a continuous variable and categorized into the following 5 groups as a measure of the TRG according to Mandard *et al* 1994 (Figure 4) [10].

### Determination of recurrence

Patients were followed up after surgery at 3-month intervals for the first 2 years, every 6-months for the next 3 years, and yearly thereafter. Patient evaluations consisted of physical examination, serum carcinoembryonic antigen (CEA), colonoscopy, abdominal ultrasound, abdominal-pelvic CT, and chest radiography according to The National Comprehensive Cancer Network (NCCN) guidelines. Other examinations such as abdominal-pelvic MRI, biopsy or operative resection, were performed for symptomatic patients if necessary. Data on whether and when the patients developed local or distant recurrence were obtained from inpatient and outpatient records. DFS was measured from the date of operation to the first local recurrence or distant metastasis. The end point of the follow-up was June 1st, 2016.

### Statistical analysis

The method we developed combining ypT stage and TRG was named M-TTRG classification. We used such method to derive a novel prognostic factor to stage primary tumors after NCRT. DFS was estimated as the endpoint of the patients in this study. Kaplan-Meier survival estimates were plotted and *P* values were assessed using log-rank tests. Hazard ratio (HR) and 95% confidence intervals (CI) were calculated using Cox proportional hazard models. The ypT stage and TRG were two factors associated with 3-year DFS, with β-coefficients of 0.368 and 0.604, respectively. We applied a linear weight to ypT stage and TRG classifications, and M-TTRG was calculated by the sum of ypT multiplied by 0.368 and TRG multiplied by 0.604. Adjacent groups ranked by M-TTRG values were grouped into five classes to get a relatively reliable result. To measure the discrimination improvement of M-TTRG classification compared with the standard ypT stage metric, we plotted receiver operating characteristic curves (ROC) and calculated the corresponding area under the curve (AUC). *P* < 0.05 was considered as statistically significant. All the data were analyzed using the Statistical Package for the Social Sciences (SPSS) version 19.0 for Windows (SPSS Inc., Chicago, IL, USA).
