# Correction: The combination of early treatment response and ypT stage is a novel metric to stage rectal cancer patients treated with neoadjuvant chemoradiotherapy

**DOI:** 10.18632/oncotarget.26723

**Published:** 2019-02-19

**Authors:** Jian Cui, Lin Yang, Lei Guo, Yongfu Shao, Dongfeng Tan, Ni Li, Haizeng Zhang

**Affiliations:** ^1^ Department of Colorectal Surgery, National Cancer Center/Cancer Hospital, Chinese Academy of Medical Sciences and Peking Union Medical College, Beijing, China; ^2^ Department of Pathology, National Cancer Center/Cancer Hospital, Chinese Academy of Medical Sciences and Peking Union Medical College, Beijing, China; ^3^ Department of Pathology and Laboratory Medicine, The University of Texas M. D. Anderson Cancer Center, Houston, TX, USA; ^4^ National Office for Cancer Prevention and Control, National Cancer Center/Cancer Hospital, Chinese Academy of Medical Sciences and Peking Union Medical College, Beijing, China

**This article has been corrected:** The correct author and affiliation information is given below:

**Jian Cui**^**1**^**, Lin Yang**^**2**^**, Lei Guo**^**2**^**, Yongfu Shao**^**1**^**, Dongfeng Tan**^**3**^**, Ni Li**^**4**^ **and Haizeng Zhang**^**1**^

^**1**^ Department of Colorectal Surgery, National Cancer Center/Cancer Hospital, Chinese Academy of Medical Sciences and Peking Union Medical College, Beijing, China

^**2**^ Department of Pathology, National Cancer Center/Cancer Hospital, Chinese Academy of Medical Sciences and Peking Union Medical College, Beijing, China

^**3**^ Department of Pathology and Laboratory Medicine, The University of Texas M. D. Anderson Cancer Center, Houston, TX, USA

^**4**^ National Office for Cancer Prevention and Control, National Cancer Center/Cancer Hospital, Chinese Academy of Medical Sciences and Peking Union Medical College, Beijing, China

Original article: Oncotarget. 2017; 8:37845-37854. https://doi.org/10.18632/oncotarget.14708

